# Convalescent patient-derived monoclonal antibodies targeting different epitopes of E protein confer protection against Zika virus in a neonatal mouse model

**DOI:** 10.1080/22221751.2019.1614885

**Published:** 2019-05-25

**Authors:** Xuefeng Niu, Lingzhai Zhao, Linbing Qu, Zhipeng Yao, Fan Zhang, Qihong Yan, Shengnan Zhang, Renshan Liang, Peihai Chen, Jia Luo, Wei Xu, Huibin Lv, Xinglong Liu, Hui Lei, Changhua Yi, Pingchao Li, Qian Wang, Yang Wang, Lei Yu, Xiaoyan Zhang, L. Aubrey Bryan, Edgar Davidson, j. Benjamin Doranz, Liqiang Feng, Weiqi Pan, Fuchun Zhang, Ling Chen

**Affiliations:** a State Key Lab of Respiratory Disease, the First Affiliated Hospital of Guangzhou Medical University, Guangzhou, People’s Republic of China; b Guangzhou 8th People’s Hospital of Guangzhou Medical University, Guangzhou, People’s Republic of China; c Guangdong Laboratory of Computational Biomedicine, Chinese Academy of Sciences, Guangzhou Institutes of Biomedicine and Health, Guangzhou, People’s Republic of China; d Institute of Physical Science and Information Technology, Anhui University, Hefei, People’s Republic of China; e University of Chinese Academy of Science, Beijing, People’s Republic of China; f Integral Molecular, Philadelphia, PA, USA

**Keywords:** Zika virus, monoclonal antibody, animal model, neutralizing epitopes, therapeutics

## Abstract

The Zika virus (ZIKV) outbreak and its link to microcephaly triggered a public health concern. To examine antibody response in a patient infected with ZIKV, we used single-cell PCR to clone 31 heavy and light chain-paired monoclonal antibodies (mAbs) that bind to ZIKV envelope (E) proteins isolated from memory B cells of a ZIKV-infected patient. Three mAbs (7B3, 1C11, and 6A6) that showed the most potent and broad neutralization activities against the African, Asian, and American strains were selected for further analysis. mAb 7B3 showed an IC50 value of 11.6 ng/mL against the circulating American strain GZ02. Epitope mapping revealed that mAbs 7B3 and 1C11 targeted residue K394 of the lateral ridge (LR) epitope of the EDIII domain, but 7B3 has a broader LR epitope footprint and recognizes residues T335, G337, E370, and N371 as well. mAb 6A6 recognized residues D67, K118, and K251 of the EDII domain. Interestingly, although the patient was seronegative for DENV infection, mAb 1C11, originating from the VH3-23 and VK1-5 germline pair, neutralized both ZIKV and DENV1. Administration of the mAbs 7B3, 1C11, and 6A6 protected neonatal SCID mice infected with a lethal dose of ZIKV. This study provides potential therapeutic antibody candidates and insights into the antibody response after ZIKV infection.

## Introduction

Zika virus (ZIKV) is a member of the *Flaviviridae* family which includes dengue virus (DENV), Japanese encephalitis virus (JEV), yellow fever virus (YFV), West Nile virus (WNV), and tick-borne encephalitis virus (TBEV) [1,2]. ZIKV is mainly transmitted by Aedes mosquitoes but can also spread through sexual contact, blood transfusions, or via mother-to-child transmission during pregnancy [3,4]. ZIKV was first discovered in Africa in 1947 [5] and was confined within the equatorial zone of Africa and Asia until the 2007 outbreak in Yap Island, which was then transmitted to French Polynesia and other Southern Pacific islands in 2013 [1,6]. It is believed that the adaptation and infectivity of ZIKV in mosquito-vectors contributed to the spread of the virus from Asia to the Americas [7]. The 2015 ZIKV outbreak and associated increase in microcephaly cases in Brazil increased global awareness [8]; to date, more than 84 countries have reported ZIKV infections [9]. It is known that ZIKV can cross the placental barrier, leading to fetal microcephaly, and can cause neurological complications in adults, such as Guillain-Barré syndrome [10–12]. Currently, there are no approved drugs or vaccines to mitigate the risk of ZIKV infection.

The ZIKV surface is formed by 180 copies of each envelope (E) glycoprotein and associated membrane (M) protein [13,14]. E proteins are arranged as dimers, with three parallel dimers connected to form a raft, and with 30 rafts covering the viral surface [15]. The E protein mediates viral entry into host cells and membrane fusion and is the major target for neutralizing antibodies and vaccine immunogens [16]. The flavivirus E ectodomain consists of three distinct domains, EDI, a 9-stranded beta-barrel that acts as a bridge between EDII and EDIII [17]; EDII, a finger-like structure that is responsible for the dimerization of soluble E protein monomers and viral fusion [18]; and EDIII, an immunoglobulin-like segment that is involved in host cell receptor recognition and viral fusion [19,20].

In recent years, a number of neutralizing antibodies (nAbs) have been isolated from individuals infected with ZIKV [21–25]. These nAbs mainly recognize EDII, EDIII, and tertiary or quaternary epitopes that constitute E ectodomains. Although EDIII-targeted antibodies represent a relatively small population of E protein-binding antibodies, their presence is associated with serum neutralizing activity against ZIKV [21,25]. Among these nAbs, EDIII-targeted antibodies and EDII/E-dimer epitope (EDE)-targeted antibodies showed the most potent neutralization activities. In this study, we cloned and characterized E-targeted monoclonal antibodies (mAbs) from a Chinese patient who returned to China from a visit to Venezuela. Selected mAbs were evaluated for their neutralizing activities *in vitro* and *in vivo* via a ZIKV-infected neonatal severe combined immunodeficiency (SCID) mouse model.

## Materials and methods

### Human subject and peripheral blood cell isolation

The patient was a 28-year-old male who returned from Venezuela in February 2016. He was hospitalized in Guangzhou 8th People’s Hospital (Guangzhou, China). ZIKV RNA was detected in serum, saliva, and urine samples by RT-PCR. The patient manifested relatively mild symptoms including fever, rash, sore throat, and fatigue, and recovered and was discharged approximately 3 weeks after the onset of symptoms with no detectable ZIKV. The patient tested serologically negative for DENV1–4 infection using an NS1-based ELISA kit (Euroimmun, Lubeck, Germany), indicating that the patient had no previous exposure to DENV1–4 before infection with ZIKV [25,26].

### Single B cell sorting, RT-PCR, sequencing, and cloning

Freshly isolated peripheral blood mononuclear cells (PBMCs) were stained with a cocktail of antibodies including anti-human CD20-FITC (Invitrogen, Carlsbad, CA), IgG-APC-H7/CD3-Pacific Blue/CD27-PerCP-Cy5.5 (BD Biosciences, Franklin Lakes, NJ), and anti-HIS-PE (Miltenyi Biotec, Bergisch Gladbach, Germany). For antigen-specific memory B cells, we used ZIKV E protein (Cat. no. 40543-V08B4; Sino Biological Inc., Beijing, China) as a probe. After washing, CD3^−^CD20^+^ CD27^+^IgG^+^HIS^+^ memory B cells were sorted using a multi-laser AriaII sorter. Individual B cells were sorted into 96-well PCR plates containing 20 µL lysis buffer per cell. The lysis buffer contained 0.25 µL RNasin inhibitor (Promega, Madison, WI), 5 µL 5X first-strand buffer, 1.25 µL 0.1 M DTT, and 0.0625 µL IGEPAL (Sigma-Aldrich, St. Louis, MO). PCR plates with sorted cells were frozen on dry ice and then stored at −80°C or subjected to reverse transcription. RT-PCR and cloning into expression vectors was performed as previously described [27]. Briefly, 1 µL random hexamers (150 ng/µL; Promega), 2 µL dNTPs (each at 10 mM), and 0.5 µL SuperScript III (Invitrogen) were added to each well, followed by incubation at 42°C for 1 h. The IgG heavy and light chain variable regions were amplified independently by nested PCR [28]. First round PCR was performed using 2 µL cDNA directly following reverse transcription, with HotStart Taq Plus DNA polymerase (Qiagen, Hilden, Germany) and the primer mix. The PCR programme was initiated by 5 min incubation at 94°C, followed by 50 cycles of 94°C for 30s, 55°C (first round) or 60°C (second round) for 30s, and 72°C for 1 min, followed by 72°C for 10 min before cooling to 4°C. Using 2% agarose gels, the PCR products were evaluated, excised from the gel (approximately 500 bp for the heavy chain and 450 bp for kappa and lambda chains), and sent for Sanger sequencing after purification. Human antibody sequences were analysed using IMGT/V-QUEST (http://www.imgt.org/) [29]. Full-length IgG1 was expressed by co-transfecting HEK-293 T cells with equal amounts of paired heavy and light chain plasmids based on the backbone of the pCI-neo vector [30]. Culture media were harvested four days after transfection and purified using protein A agarose (GE healthcare, Chicago, IL).

### Viruses and recombinant proteins

ZIKV particles were successfully isolated from the infected patient’s blood plasma or urine. The virus was passaged once in suckling mouse brains and cultured in Vero cells to prepare stocks, which were stored at −80°C before use. DENV1 (Hawaii strain), DENV2 (New Guinea-C strain), DENV3 (H87 strain), and DENV4 (H241 strain) were prepared in Vero cells. Recombinant ZIKV E and EDIII protein (Cat. no. 40543-V08B4 and 40543-V08H, respectively) were purchased from Sino Biological Inc. The E protein was derived from ZIKV strain SPH2015(KU321639), isolated from Brazil in 2015. DENV1 EDIII protein (Cat. no. 40531-V08B), DENV2 EDIII protein (Cat. no. 40471-V08Y3), and DENV4 E protein (Cat. no. 40533-V08B2) were also purchased from Sino Biological Inc.

### Enzyme-linked immunosorbent assay (ELISA)

Nunc MaxiSorp plates (Thermo Fisher Scientific, Waltham, MA) were coated with ZIKV E or E domain III protein (1 µg/mL) and incubated at 4°C overnight. After blocking for 2 h, the plates were washed six times with phosphate-buffered saline (PBS) and transfected with antibody supernatants at a 1:2 dilution with blocking buffer or a serial dilution of purified mAbs. Secondary antibody (goat anti-human IgG; ab6858; Abcam, Cambridge, UK) was applied at a 1:5000 dilution in blocking solution and then the plates were incubated at room temperature for 1 h, and TMB substrate. Absorbance values were determined at 450 nm using a BioTek plate reader (BioTek Instruments, Winooski, VT).

### Neutralization assay

Neutralization activity of purified antibodies was measured using a flow cytometry-based neutralization assay with Vero cells as previously reported [24,31] with minor modifications. Briefly, 2 × 10^5^ cells were plated in a 24-well plate 24 h before the experiment. Purified human mAbs were serially diluted in DMEM (Gibco; Thermo Fisher Scientific) supplemented with 1% feta bovine serum (FBS; Gibco) and incubated with ZIKV (5 × 10^3^ PFU**)** for 1 h at 37°C under 5% CO_2_. The cells were then incubated with 300 µL of the mixture for 1 h at 37°C under 5% CO_2_, after which 1 mL/well MEM medium containing 10% FBS was added and incubated for another 40 h. ZIKV-infected and uninfected cells were used as positive and negative control, respectively. Cells were then trypsinized, fixed, and permeabilised with fixation and permeabilization solution (BD Biosciences) on ice for 20 min, followed by staining with 4G2 antibody (2 µg/mL) diluted with 1X Perm/Wash buffer and staining with anti-mouse IgG FITC (1:100 in 1X Perm/Wash buffer) on ice for 30 min. After washing, the percentage of E-positive cells was measured using BD FACScanto II (BD Biosciences). The antibody dilution that neutralized 50% of the viruses (half-maximal neutralizing inhibitory concentration; IC50) was calculated by nonlinear, dose-response regression analysis with GraphPad Prism version 6.0 (GraphPad Software Inc., La Jolla, CA).

### Shotgun mutagenesis epitope mapping

Epitope mapping was performed by shotgun mutagenesis as previously described [32]. ZIKV prM-E protein expression constructs (based on ZIKV strain SPH2015) were subjected to high-throughput alanine scanning mutagenesis to generate a comprehensive mutation library [22,23]. Each residue within prM-E was changed to alanine, with alanine codons mutated to serine. In total, 672 ZIKV prM-E mutants were generated (100% coverage) – which were confirmed by sequencing – and arrayed into 384-well plates. Each ZIKV prM-E mutant was transfected into HEK-293 T cells and incubated for 22 h. Cells were then fixed in 4% (v/v) paraformaldehyde (Electron Microscopy Sciences, Hatfield, PA) and permeabilised with 0.1% (w/v) saponin (Sigma-Aldrich) in PBS plus calcium and magnesium (PBS++). For mapping, cells were sequentially incubated with 1.0 µg/mL mAbs and 3.75 µg/mL AlexaFluor 488-conjugated secondary antibody (Jackson ImmunoResearch Laboratories, West Grove, PA) diluted in PBS++, 10% normal goat serum (Sigma-Aldrich), and 0.1% saponin. Cells were washed three times with PBS++/0.1% saponin, twice with PBS, and then mean cellular fluorescence was recorded using a high-throughput flow cytometer (HTFC; IntelliCyt, Albuquerque, NM). Antibody reactivity against each prM-E mutant relative to the wild-type (WT) protein was calculated by subtracting the signal from mock-transfected controls and normalizing to the signal from WT prM-E-transfected controls.

### Binding competition assay

Binding competition between mAbs 7B3, 1C11, and 6A6 and five other EDIII-targeted antibodies was determined using a real-time, label-free, bio-layer interferometry assay on an Octet RED96 biosensor (ForteBio, Fremont, CA) as previously described [24]. The experiment was performed at 30°C in PBS buffer with shaking at 1,000 rpm. Ni-NTA biosensors (ForteBio) were first loaded with 4 μg/mL His-tagged-E protein for 300 s and then associated with the first mAb (7B3, 7F4, 8D10, 1C11, 6A6, 6B6, 6D6, or 6F1) for 900 s. An irrelevant mAb, 2D1, was used as negative control and PBS was used as blank solution. The biosensors were then dipped into the second mAb and incubated in the presence of the first mAb. The capacity of additional binding was monitored by measuring further shifts for 300 s. All mAbs were evaluated at concentration of 150 nM, except for 6B6 (700 nM), 6D6 (900 nM), and 6F1 (900 nM), for saturation measurement. The Ni-NTA biosensors were regenerated with 10 mM glycine-HCl (pH 1.7; GE Healthcare) and re-charged with 10 mM NiCl_2_. The response of mAb binding to the E protein was compared and the data were processed using BIAevaluation software (Biacore, Uppsala, Sweden).

### Mouse protection experiments

The use of pregnant SCID and suckling mice in this study was carried out in strict compliance with the Association for the Assessment and Accreditation of Laboratory Animal Care. The experimental protocol was approved by the Guangzhou Institute of Biomedicine and Health (GIBH) Institutional Animal Care and Use Committee. SCID Beige mice were purchased from Beijing Vital River Laboratory Animal Technology Co., Ltd. (Beijing, China). One-day-old suckling mice (*n* = 5–8) were intraperitoneally inoculated with ZIKV at 1.2 × 10^4^ PFU. ZIKV mAbs were administered as a single dose 24 h after virus infection. Mouse brain, spleen, and serum samples were then collected 15 days after virus infection for RNA extraction (74804; Qiagen). ZIKV RNA detection was determined by one-step qRT-PCR (204243; Qiagen) on a CFX96 Real-Time PCR System (Bio-Rad Laboratories, Hercules, CA) using published primers and conditions. Briefly, 4 µg RNA together with a mixture of 7.5 µL SYBR Green, 0.15 µL RT-mix, and 0.25 µL each of forward and reverse primers were placed in 96-well PCR plates in 15 µL reaction volumes. Amplification was performed at 50°C for 30 min, 95°C for 15 min, followed by 44 cycles of 94°C for 15 s, 55°C for 30 s, and 72°C for 30 s. Viral load was expressed on a log10 scale as viral RNA copies per µg after comparison with a standard curve. Three replicates were conducted for each sample.

### Statistical analysis

Flow cytometric data were analysed using FlowJo version 7.6 (Tree Star Inc., Ashland, OR). The EC50 (half-maximal effective binding concentration) and IC50 values obtained from the ELISA assay of ZIKV E/EDIII binding activity to mAbs and the neutralization assay, respectively, were calculated using a dose-response inhibition model and sigmoidal curves were generated using GraphPad Prism version 6.0 (GraphPad Software Inc.). Statistical significance of the difference in viral loads between groups was determined using one-way ANOVA. *p* values < 0.05 were considered statistically significant.

## Results

### Cloning and characterization of ZIKV E-targeted mAbs from a convalescent ZIKV-infected patient

**Figure 1.**
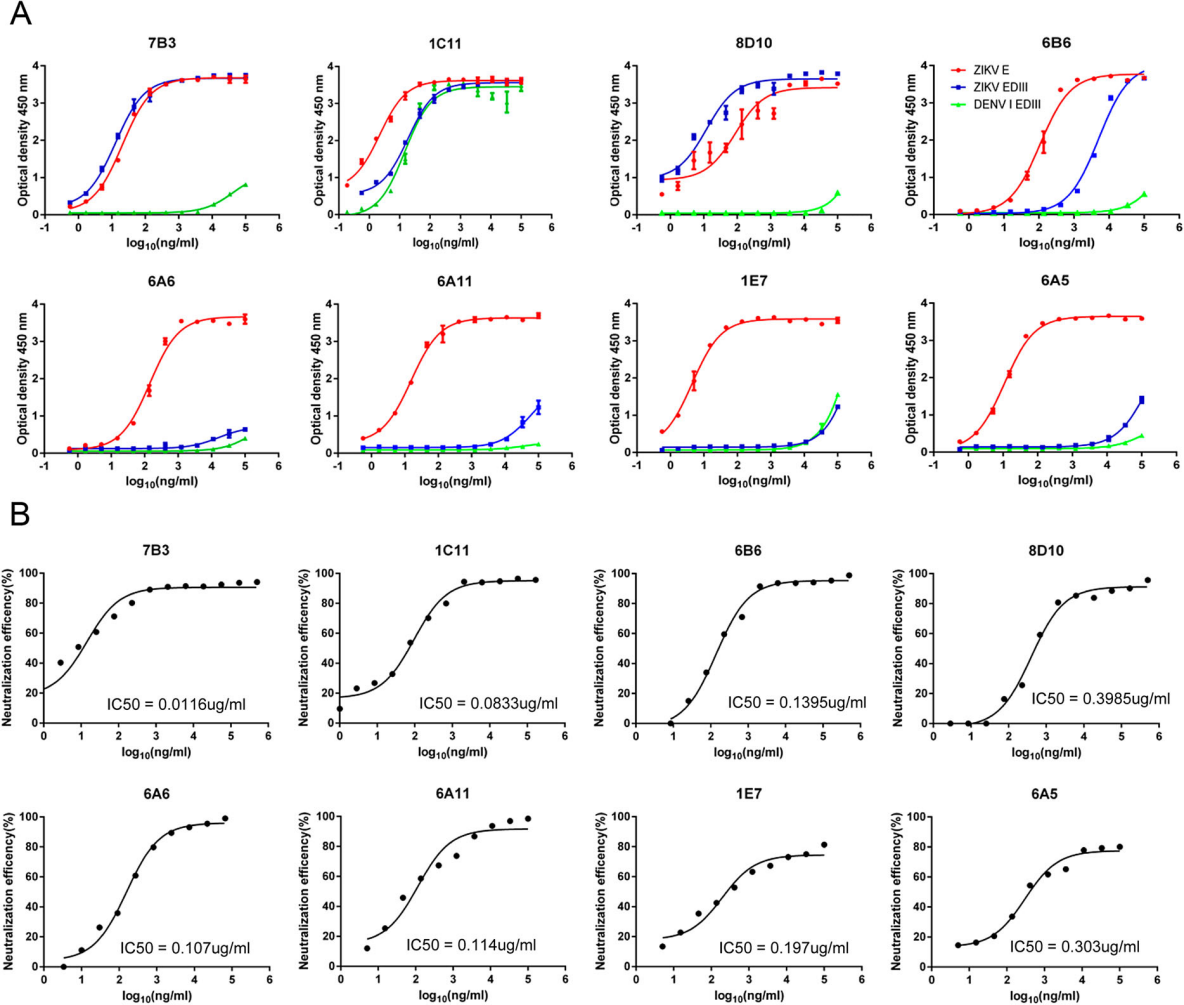
Binding and neutralization activities of representative E-targeted mAbs. (A) Binding activities of E-targeted mAbs to ZIKV E, EDIII, and DENV1 EDIII protein. (B) Neutralizing activity of E-targeted mAbs against ZIKV GZ02 strain. Serial dilutions of mAbs were tested for neutralizing activity against ZIKV GZ02 using a FACS-based neutralization test (FNT). Data are representative of two independent experiments.

To clone E-targeted mAbs from a ZIKV-infected patient, we used recombinant ZIKV E protein as a probe to isolate E-specific memory B cells from a Chinese patient that had returned from Venezuela and was identified as infected with ZIKV during border entry. Thirty-one mAbs that bind to E protein were successfully cloned 64 days after the onset of symptoms. We first used a FACS-based neutralization assay to screen neutralizing activities in the cultured media of HEK-293 cells transfected with expression plasmids for each heavy and light chain of the mAbs. Eight mAbs with the best neutralizing activities and binding to E protein were selected for further analysis. The binding activities of these eight mAbs to ZIKV E, EDIII, and DENVI EDIII proteins were assessed (Figure 1 and Table 1). mAbs 7B3, 1C11, 8D10, and 6B6 bound both ZIKV E and EDIII, while mAbs 6A6, 6A11, 1E7, and 6A5 bound E protein but not EDIII (Figure 1(A)). mAbs 7B3 and 1C11 showed strong binding activities to ZIKV E and EDIII protein in the nanomolar range.

**Table 1.** Binding and neutralizing activities of E-targeted mAbs.

EC50/IC50 (µg/mL)*	7B3	1C11	6B6	8D10	6A6	6A11	1E7	6A5
ZIKV E-binding	0.0022	0.002	0.107	0.0864	0.0147	0.015	0.044	0.011
ZIKV EDIII-binding	0.0128	0.0186	5.02	0.0121	>75	>75	>75	>75
DENV1 EDIII-binding	41.6	0.0146	>75	>75	>75	>75	>75	>75
DENV2 EDIII-binding	>75	>75	>75	>75	>75	>75	>75	>75
DENV3-binding	>75	>75	>75	>75	>75	>75	>75	>75
DENV4 E-binding	>75	>75	>75	>75	>75	>75	>75	>75
GZ02 neutralization	0.0116	0.0833	0.1395	0.3985	0.107	0.114	0.197	0.303
PRVABC59 neutralization	0.0448	0.0986	ND	ND	0.292	ND	ND	ND
MR766 neutralization	0.176	0.805	ND	ND	0.41	ND	ND	ND
DENV1 neutralization	>75	1.72	ND	ND	>75	ND	ND	ND

*EC50 of mAb binding to ZIKV E and EDIII were measured, as well as the IC50 of mAbs neutralizing the ZIKV strains GZ02, PRVABC59, and MR766. ND, not determined.

We then measured the neutralization activities of purified mAbs against the ZIKV GZ02 strain. ZIKV GZ02 has the same genomic sequence as the current circulating ZIKV strain in South America and was the same as the ZIKV strain isolated from the patient [33]. Three mAbs, 7B3, 1C11, and 6A6, showed the best neutralization activities with IC50 values of 11.6, 83.3, and 107 ng/mL, respectively; thus, we further analysed these mAbs for epitope identification and in an animal model (Figure 1(B)). We also tested the neutralizing activities of these three mAbs against two other ZIKV isolates, MR766 (the first ZIKV isolated from the Zika forest in Uganda) [5] and PRVABC59 (a ZIKV isolated from Puerto Rico). All three antibodies cross-neutralized MR766 and PRVABC59 (Table 1). Intriguingly, one of the mAbs, 1C11, showed binding to DENV1 EDIII protein but not to other DENV EDIII proteins. The ED50 of 1C11 with DENV EDIII was 14.6 ng/mL, which was comparable to ZIKV EDIII binding (18.6 ng/mL). We then measured the neutralization activity of these three representative mAbs against DENV1 and found that mAbs 7B3 and 6A6 showed no DENVI EDIII cross-reactivity and failed to neutralize DENVI virus at 75 µg/mL, whereas 1C11 showed potent neutralizing activity at an IC50 of 1.72 µg/mL (Table 1).

### Neutralizing mAbs recognized either the EDIII or EDII lateral ridge (LR)

**Figure 2.**
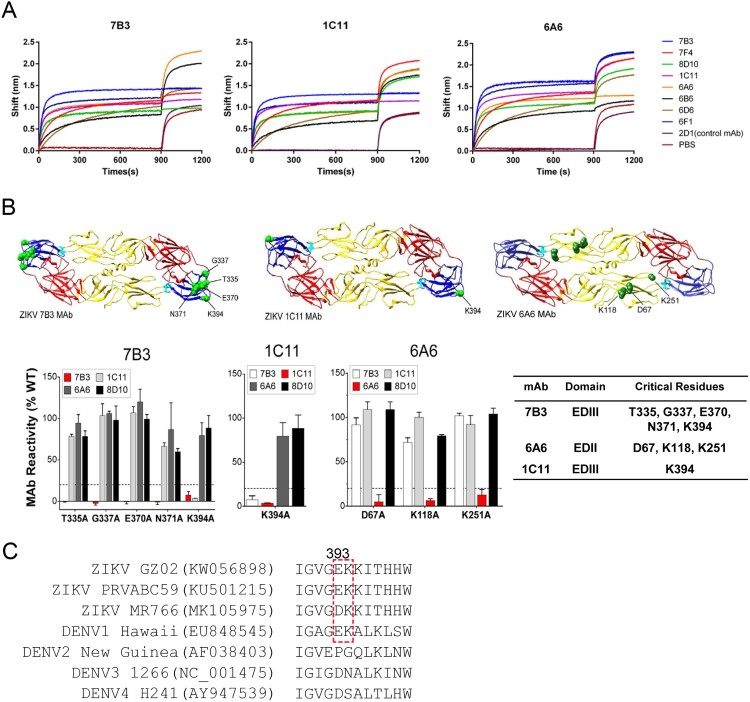
Competition study and epitope mapping of representative E-targeted mAbs 7B3, 1C11, and 6A6. (A) Competition between mAbs 7B3, 1C11, and 6A6 and other EDIII-targeted antibodies was determined by an Octet competition assay. Ni-NTA biosensors loaded with E protein were first saturated for 900 s with the indicated mAbs (7B3, 7F4, 8D10, 1C11, 6A6, 6B6, 6D6, or 6F1). An irrelevant mAb, 2D1, was used as negative control and PBS as blank solution. The capacity for additional binding was monitored for 300 s by measuring further shifts after incubating with the second antibodies 7B3, 1C11, or 6A6. The red dotted vertical line represents the second mAb loading time. (B) Critical amino acid residues recognized by mAbs 7B3, 6A6, and 1C11. Epitope mapping was performed by measuring mAb binding to a comprehensive library of alanine scan mutations at every residue of ZIKV prM-E protein (SPH2015 strain). Identified critical residues for mAb binding are shown via a ribbon diagram of ZIKV E. mAb binding reactivity for each alanine mutant is expressed as percentage of reactivity of mAb with ZIKV prM-E. Clones with reactivity at 30% lower than that of wild-type ZIKV prM-E were identified as critical residues for binding. (C) Sequence alignment of amino acids near residue 394 of ZIKV and DENV E proteins. The virus strains and GenBank access numbers are indicted.

EDIII has been recognized as a major target site for nAbs that bind to the same or different epitope region. We used a bio-layer interferometry (BLI) competition assay to probe whether our E-targeted mAbs bind to the same epitope region. A His-tagged E protein, captured to a Ni-NTA biosensor, was first saturated with one mAb, after which competitive binding of a second mAb was assessed. The binding competition between mAbs 7B3, 1C11, and 6A6 and five other ZIKV E-targeted mAbs was measured (Figure 2(A)). mAbs 1C11, 8D10, 6D6, 7F4, and 6F1 but not 6A6 could block the binding of mAb 7B3 to E protein, indicating that 7B3 and these five mAbs share the same or overlapping epitopes on ZIKV EDIII. Interestingly, mAb 1C11 binding to E protein was blocked by mAb 7B3 and no other E-targeted mAb, indicating that 7B3 may bind to a broader epitope footprint on EDIII, whereas 1C11 binds to a smaller epitope region that overlaps with the binding region of 7B3.

We next mapped the detailed epitope residues for mAbs 7B3, 1C11, and 6A6 using a shotgun alanine-scanning mutagenesis library of ZIKV prM and E protein variants [22,23]. mAb 7B3 recognized an epitope region that includes residues T335, G337, E370, N371, and K394 along the LR region of EDIII. Notably, mAb 1C11 only recognized K394 on the LR region as a key residue (Figure 2(B and C)). Amino acid sequence alignment between E proteins of ZIKV and DENV revealed that residue K394 on ZIKV E protein corresponds to K385 on DENV1, which is not present on the E proteins of DENV2, DENV3, or DENV4 (Figure 2(D)). mAb 6A6 is an EDII-targeted antibody that recognized residues D67, K118, and K251 on the EDII. We confirmed that an individual mutation of these key residues reduced binding activity by more than 70% when compared with that of WT prM-E protein (Figure 2(B)).

### E-targeted neutralizing mAbs were obtained with a low level of somatic hypermutions (SHMs)

**Figure 3.**
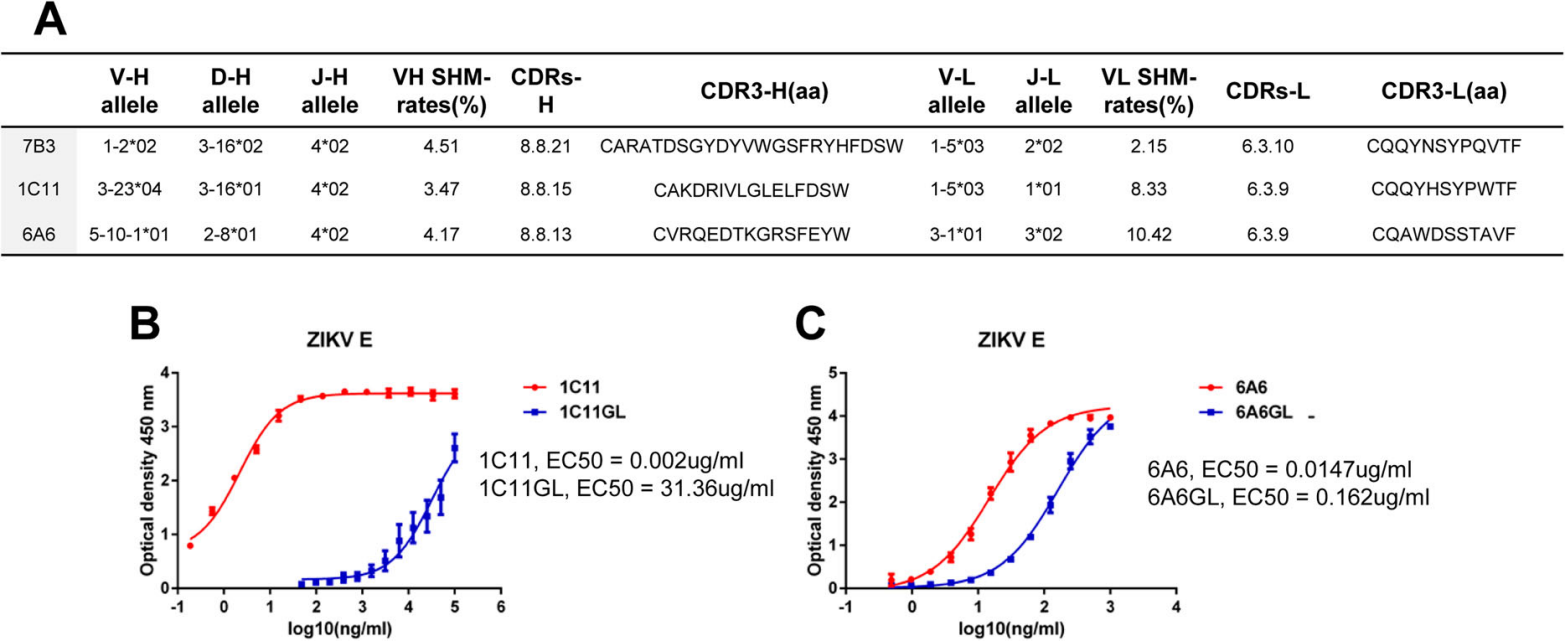
Somatic mutations of mAbs 1C11 and 6A6 are required for ZIKV E protein binding. (A) V(D)J usage in 7B3, 1C11, and 6A6 mAbs. VH, D, and JH gene usage and the level of somatic mutations in VH, CDRHs, and HCDR3 amino acid sequence. (B) Binding activity analysis of 1C11 and 1C11GL with recombinant ZIKV E protein was performed via ELISA. (C) Binding activity analysis of mAb 6A6 and 6A6GL with recombinant ZIKV E protein was performed via ELISA.

Analysis of the immunoglobulin heavy chains of 7B3, 1C11, and 6A6 revealed that they were derived from germlines HV1-2*02, HV3-23*04, and HV5-10-1*01, respectively (Figure 3(A)). The SHM rates of these heavy chains compared with their predicted germline sequences were relatively low, at 4.51% for 7B3H, 3.47% for 1C11H, and 4.17% for 6A6H, which is lower than that of antibodies isolated from annual trivalent inactivated influenza vaccine (TIV) donors [34] and chronic human immunodeficiency virus (HIV)-1 patients (>30%) [27,35]. Compared with their germline sequences, the SHMs of 6A6H were in the CDR1, CDR2, and CDR3 regions, whereas SHMs of 1C11H and 7B3 were found sporadically in the CDR1, FR2, CDR2, FR3, and CDR3 regions (Table 2). We paired the heavy chain predicted germline (HGL) of 1C11H (1C11HGL) with the light chain (kappa) of 1C11 (1C11 K) and similarly paired 6A6HGL with 6A6 K and expressed these heavy chain germline-reverted antibodies in HEK-293 cells. These germline-reverted antibodies showed reduced binding activities to E protein at 0.162 µg/mL for 6A6GL, which is about 10 times lower than that of mature 6A6 (0.0147 µg/mL), and 31.36 µg/mL for 1C11GL, which almost completely lost binding ability compared with that of mature 1C11 (0.002 µg/mL; Table 2). Therefore, SHMs are necessary for strong binding to E protein, but only a low level of SHMs is needed to improve binding.

**Table 2.** CDR1, FR2, CDR2, and FR3 amino acid sequence of 7B3H, 1C11H, and 6A6H with their corresponding VH germline sequences and predicted CDR3 sequences.

	CDR1*		FR2				CDR2				FR3							CDR3				
	35	36	39	40	48	55	56	59	62	63	70	71	83	85	92	100	101	105	111	119	122	125
6A6H-UCA	–	S	–	–	K	–	–	S	–	–	–	F	S	–	S	–	M	A	–	–	–	–
6A6H	–	N	–	–	R	–	–	R	–	–	–	V	A	–	G	–	V	V	–	–	–	–
1C11H-UCA	–	S	–	–	–	A	I	–	G	G	–	–	–	–	–	–	–	–	–	Y	–	–
1C11H	–	N	–	–	–	G	F	–	D	S	–	–	–	–	–	–	–	–	–	S	–	–
7B3H-UCA	T	–	M	H	–	–	–	–	–	–	K	–	–	S	–	A	–	–	Y	Y	T	Y
7B3H	I	–	I	N	–	–	–	–	–	–	N	–	–	R	–	S	–	–	G	F	H	S

*CDR1, FR2, CDR2, FR3, CDR3, and site number are defined in the IMGT format. “–” no amino acid mutants compared with the VH germline and predicted CDR3 germline sequences. Region ranges: CDR1(35–36), FR2 (39–55), CDR2 (56–63), FR3 (70–101), and CDR3(105–125).

### E-targeted mAbs can effectively protect neonatal SCID mice from lethal ZIKV infections

**Figure 4.**
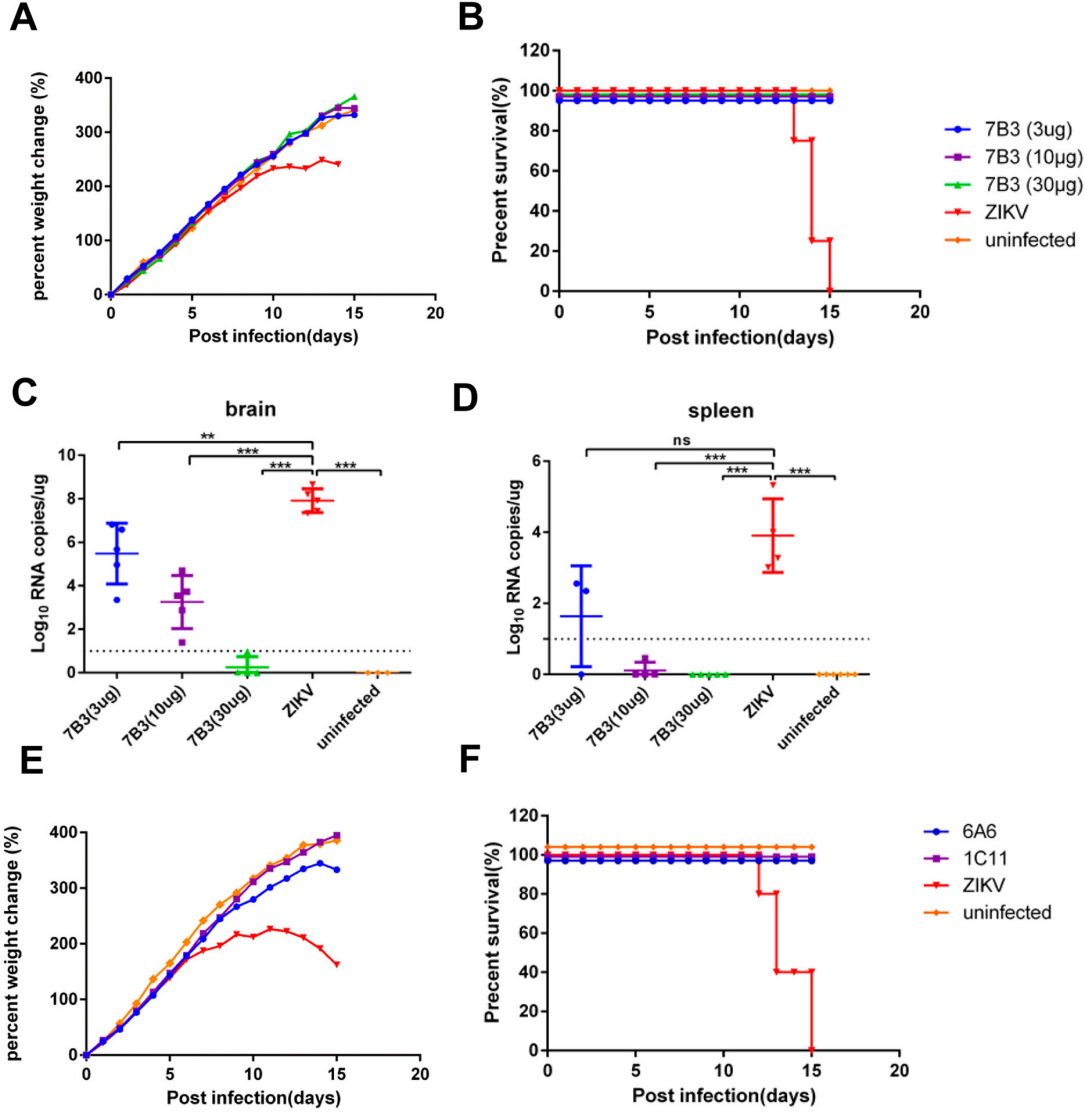
Treatment with E-targeted mAbs against lethal ZIKV infection in neonatal SCID mice. Neonatal SCID mice (*n* = 5–8) were intraperitoneally inoculated with ZIKV GZ02 1.2 × 10^4^ PFU. ZIKV mAbs were administered as a single dosage 24 h after infection. (A) Body weight changes in ZIKV-infected mice treated with mAb 7B3 at 3, 10 µg, or 30 µg/mouse. Uninfected and ZIKV-infected mice were used as controls. (B) Survival curve of ZIKV-infected and mAb 7B3-treated mice. (C) Viral loads in the brain (*n* = 5–8 per group). (D) Viral load in the spleen (*n* = 5–8). Total RNA was extracted from the homogenates of the brain and spleen. ZIKV genomic RNA was evaluated using one-step qPCR. Viral loads are expressed as the genome copy number per microgram tissue. (E) Body weight changes in ZIKV-infected mice treated with mAb 6A6 or 1C11 at 30 µg/mouse. (F) Survival curve of ZIKV-infected mice treated with mAb 6A6 or 1C11. Data are representative of two independent experiments and presented as mean ± SEM. *p < 0.05; **p < 0.01; ***p < 0.001; ns, no significance (one-way ANOVA).

To evaluate the protective effects of E-targeted mAbs, we developed a ZIKV lethal infection mouse model. Intraperitoneal infection of 1-day-old SCID neonates with 1.2 × 10^4^ PFU ZIKV GZ02 caused 100% lethality within 15 days; neonatal mice that received no treatment showed disease symptoms, including ruffled fur, trembling and shaking, and body weight loss (Figure 4(A)). Neonatal mice treated with mAb 7B3 at either 3, 10 µg, or 30 µg 24 h after infection survived the lethal infection (Figure 4(B)). Titration of viral load from brains and spleens collected 12 days after infection revealed a dose-dependent decrease in viral RNA in mice treated with mAb 7B3, whereas mice that received no treatment had much higher viral RNA levels in the brain and spleen (Figure 4(C and D)). Treatment with 30 µg mAb 7B3 resulted in the best protection, with no detectable viral RNA in the brain and spleen. We also demonstrated in subsequent experiments that ZIKV-infected neonatal SCID mice survived and did not exhibit weight loss after treatment with 30 µg of either mAb 1C11 or 6A6 (Figure 4(E and F)). In a separate experiment, an unrelated mAb, 2G11, which is specific for H7N9 influenza virus, showed no protective effects on ZIKV-infected neonatal SCID mice (data not shown). Therefore, both EDIII-targeted and EDII-targeted mAbs can effectively protect neonatal SCID mice from lethal ZIKV infections.

## Discussion

The emergence of ZIKV in South America has raised a global health concern due to the link between ZIKV infection and microcephaly in infants and Guillain-Barré syndrome in adults. The search for and development of vaccines and therapeutics to prevent and control ZIKV infection are thus necessary. For example, nAbs have been found effective in combating emerging viruses, such as the Middle East respiratory syndrome coronavirus (MERS-CoV), Ebola virus, and influenza virus [30,36–38].

In the present study, we cloned ZIKV E protein-binding mAbs from the memory B cells of a ZIKV-infected Chinese patient and selected three mAbs for further study. We characterized the ZIKV E protein epitopes recognized by these mAbs and analysed the SHM pattern. Two of the most potent mAbs, 7B3 and 1C11, are EDIII-targeted and showed neutralizing activities against three different ZIKV strains, including the South American circulating strain (GZ02), African strain (MR766), and American strain (PRVABC59). mAb 7B3 recognized several residues in the LR region of EDIII, and an individual mutation in these residues led to a > 70% decrease in binding activity compared with that of WT prM-E protein. A previous study also demonstrated that an E370 K mutation alone abolished the neutralizing activity of the LR-targeted mAb ZKA190 [39]. Meanwhile, the heavy and light chains of mAb 1C11 were derived from the VH3-23 and VK1-5 germlines, respectively. It has been reported that ZIKV mAbs with VH3-23/VK1-5 paired antibodies are present in five of six people that were sequentially infected with DENV1 and ZIKV; thus, these mAbs were determined as recurrent antibodies that recognize both ZIKV and DENV1 viruses [21]. Our findings, along with observations by other studies [40], suggested that VH3-23 may be preferentially enlisted in response to ZIKV and DENV infections. It is interesting to note that both mAb 7B3 and 1C11 recognized K394 in the LR region of ZIKV EDIII. mAbs reported by others, such as Z004 and Z006, also recognized K394 on ZIKV EDIII or K385 on DENV1 EDIII [21]. Although mAb 1C11 neutralized the African ZIKV strain MR766, another reported mAb, ZIKV-116, that also recognized K394 of the ZIKV E protein and neutralized the H/PF/2013 strain, failed to neutralize the MR766 strain [22]. This difference may because the current ZIKV strains GZ02 and PRVABC59 possess E393 in the EDIII region, whereas African strain MR766 has D393 in the EDIII region. Therefore, K394 in LR region of EDIII appears to be a key target residue for EDIII-targeted antibodies to exert neutralizing activities. mAbs that recognized K394 showed potent *in vitro* neutralizing activity, supporting the notion that residue K394 is a hotspot for effective neutralizing activity of EDIII-targeted mAbs [21,22,24,41,42].

It is important to note that most reported E-targeted neutralizing mAbs are EDIII-targeted, whereas only a few neutralizing mAbs are EDII-targeted. mAbs showing broad neutralization activities against flaviviruses have been reported to recognize the EDII targeting either the fusion loop epitope (FLE) or EDE regions [33,43]. Antibodies targeting the FLE are conserved among flaviviruses and can bind to ZIKV E protein. It has been reported that FLE-targeted antibodies show poor neutralizing activities but have strong infection enhancement *in vitro* [44]. Meanwhile, antibodies targeting the EDE show potent neutralizing activities against ZIKV and can protect against ZIKV infection in mouse and rhesus macaque models [45,46]. Notably, mAb 6A6 in our study showed potent neutralizing activities against all three tested ZIKV strains and is likely an EDII-targeted mAb. It also recognized residues D67, K118, and K251 and is similar to the reported EDE-targeted antibody ZIKV-117, which recognizes D67, K118, and Q89 [22]. Both mAbs recognize the same two residues in the EDII region. Structural analysis indicated that mAb ZIKV-117 is cross-linked with E monomers within dimers and neighbouring dimers in the ZIKV particle [22,47]. Therefore, mAb 6A6 may not exhibit the same interaction with the virus particle as ZIKV-117. Recently, another EDE-targeted mAb, ZIKV-195, was reported to neutralize multiple ZIKV strains and recognize residues D67, M68, R73, and K251 in the EDII region [48]; both mAb 6A6 and ZIKV-195 recognize residues D67 and K251. Notably, mAb 6A6, ZIKV-117, and ZIKV-195 were all isolated from memory B cells of ZIKV convalescent patients. It is possible that residues D67, K118, and K251 are hotspots for EDE-targeted neutralizing antibodies. Future structural biology analysis is required to confirm if mAb 6A6 is indeed an EDE-targeted antibody.

Antigen-specific B cells undergo a process termed SHM to increase antigen affinity. Interestingly, the neutralizing mAbs 7B3, 1C11, and 6A6 had relatively low SHM rates in their VH genes, which is much lower than the antibodies isolated from annual TIV vaccine donors [34] and chronic HIV-1 patients [27,35]. It is possible that when a person is exposed to ZIKV infection, E-targeted antibodies that require low SHM rates to bind and neutralize ZIKV were generated. Even with relatively low SHM rates, these limited mutations appeared essential for conferring binding activities. When the mAbs 1C11 and 6A6 heavy chains were reverted to their predicted germline sequence and paired with their respective mature 1C11 and 6A6 light chains, binding activity to ZIKV E protein was found almost completely impaired and 10 times lower than that of mature 6A6, respectively. Therefore, a few mutations are sufficient but necessary to confer neutralizing activities against ZIKV infection.

In addition to evaluating the neutralizing activities of representative mAbs in inhibiting ZIKV infection in cultured cells, we also tested the protective efficacy of these mAbs in a neonatal SCID mouse model. ZIKV infection in WT mice does not result in disease, whereas suckling WT mice are susceptible to infection [49,50]. Mice lacking interferon signalling, such as A129 (type I IFNAR KO) [49], interferon regulatory factor (IRF) 3/5/7 triple KO [49], and AG129 (type 1 and type2 IFN KO) [51], are susceptible to ZIKV infection with detectable ZIKV in the brain, spinal cord, and testes; these mice died within 5–10 days post-infection. In this study, we developed a SCID Beige suckling mouse model for evaluating the protective ability of mAbs against ZIKV infection. SCID Beige mice are deficient in T, B, and NK cells and are thus more suitable for evaluating the net effect of anti-ZIKV mAbs. ZIKV infection of SCID Beige suckling mice led to neurological symptoms and high viral loads in the brain and spleen, which was eventually lethal. All three mAbs tested (EDIII-targeted mAbs 7B3 and 1C11 and EDII-targeted mAb 6A6) showed protective effects in ZIKV-infected SCID mice. Therefore, nAbs against ZIKV cloned from convalescent patients have the potential to be further developed for treating ZIKV infection.

There is an opinion that E-targeted mAbs may mediate antibody-dependent enhancement (ADE) of virus infection. We found that mAb 7B3 enhanced ZIKV infection in K562 cells at a very low concentration of 10 ng/mL. However, we believe that ADE is not a critical concern because the concentration of mAbs used for treatment are much higher. Nevertheless, engineering of the mAb Fc fragment to minimize Fc gamma receptor-mediated infection would be beneficial in improving the practical usage of these neutralizing mAbs. Overall, our study findings provided insights into the antibody response after ZIKV infection and demonstrated the potential of mAbs in ZIKV treatment.

## References

[1] Lazear HM, Diamond MS, Pierson TC. Zika virus: new clinical syndromes and its emergence in the Western Hemisphere. J Virol. 2016;90:4864–4875. doi: 10.1128/JVI.00252-16 26962217 PMC4859708

[2] Gatherer D, Kohl A. Zika virus: a previously slow pandemic spreads rapidly through the Americas. J Gen Virol. 2016;97:269–273. doi: 10.1099/jgv.0.000381 26684466

[3] Joguet G, Mansuy J-M, Matusali G, et al. Effect of acute Zika virus infection on sperm and virus clearance in body fluids: a prospective observational study. Lancet Infect Dis. 2017;17:1200–1208. doi: 10.1016/S1473-3099(17)30444-9 28838639

[4] Pierson TC, Diamond MS. The emergence of Zika virus and its new clinical syndromes. Nature. 2018;560:573–581. doi: 10.1038/s41586-018-0446-y 30158602

[5] Dick G, Kitchen S, Haddow A. Zika virus (I). Isolations and serological specificity. Trans R Soc Trop Med Hyg. 1952;46:509–520. doi: 10.1016/0035-9203(52)90042-4 12995440

[6] Hayes EB. Zika virus outside Africa. Emerg Infect Dis. 2009;15:1347–1350. doi: 10.3201/eid1509.090442 19788800 PMC2819875

[7] Liu Y, Liu J, Du S, et al. Evolutionary enhancement of Zika virus infectivity in Aedes aegypti mosquitoes. Nature. 2017;545:482–486. doi: 10.1038/nature22365 28514450 PMC5885636

[8] Lessler J, Chaisson LH, Kucirka LM, et al. Assessing the global threat from Zika virus. Science. 2016;353:aaf8160. doi: 10.1126/science.aaf8160 27417495 PMC5467639

[9] McArthur M. Zika virus: recent advances towards the development of vaccines and therapeutics. Viruses. 2017;9:143. doi: 10.3390/v9060143 PMC5490820 28608813

[10] Cao-Lormeau V-M, Blake A, Mons S, et al. Guillain-Barré syndrome outbreak associated with Zika virus infection in French Polynesia: a case-control study. The Lancet. 2016;387:1531–1539. doi: 10.1016/S0140-6736(16)00562-6 PMC5444521 26948433

[11] Schuler-Faccini L, Ribeiro EM, Feitosa IM, et al. Possible association between Zika virus infection and microcephaly - Brazil, 2015. MMWR Morb Mort Wkly Rep. 2016;65:59–62. doi: 10.15585/mmwr.mm6503e2 26820244

[12] Mlakar J, Korva M, Tul N, et al. Zika virus associated with microcephaly. N Engl J Med. 2016;374:951–958. doi: 10.1056/NEJMoa1600651 26862926

[13] Sirohi D, Chen Z, Sun L, et al. The 3.8Å resolution cryo-EM structure of Zika virus. Science. 2016;352:467–470. doi: 10.1126/science.aaf5316 27033547 PMC4845755

[14] Kostyuchenko VA, Lim EX, Zhang S, et al. Structure of the thermally stable Zika virus. Nature. 2016;533:425–428. doi: 10.1038/nature17994 27093288

[15] Zhang Y, Zhang W, Ogata S, et al. Conformational changes of the flavivirus E glycoprotein. Structure. 2004;12:1607–1618. doi: 10.1016/j.str.2004.06.019 15341726 PMC4152830

[16] Smit J, Moesker B, Rodenhuis-Zybert I, et al. Flavivirus cell entry and membrane fusion. Viruses. 2011;3:160–171. doi: 10.3390/v3020160 22049308 PMC3206597

[17] Cockburn JJ, Navarro Sanchez ME, Goncalvez AP, et al. Structural insights into the neutralization mechanism of a higher primate antibody against dengue virus. EMBO J. 2012;31:767–779. doi: 10.1038/emboj.2011.439 22139356 PMC3273384

[18] Dai L, Song J, Lu X, et al. Structures of the Zika virus envelope protein and its complex with a flavivirus broadly protective antibody. Cell Host Microbe. 2016;19:696–704. doi: 10.1016/j.chom.2016.04.013 27158114

[19] Rey F, Heinz F, Mandl C, et al. The envelope glycoprotein from tick-borne encephalitis virus at 2Å resolution. Nature. 1995;375:291–298. doi: 10.1038/375291a0 7753193

[20] Stiasny K, Kössl C, Lepault J, et al. Characterization of a structural intermediate of flavivirus membrane fusion. PLoS Pathog. 2007;3:e20. doi: 10.1371/journal.ppat.0030020 17305426 PMC1797619

[21] Robbiani DF, Bozzacco L, Keeffe JR, et al. Recurrent potent human neutralizing antibodies to Zika virus in Brazil and Mexico. Cell. 2017;169:597–609.e11. doi: 10.1016/j.cell.2017.04.024 28475892 PMC5492969

[22] Sapparapu G, Fernandez E, Kose N, et al. Neutralizing human antibodies prevent Zika virus replication and fetal disease in mice. Nature. 2016;540:443–447. doi: 10.1038/nature20564 27819683 PMC5583716

[23] Stettler K, Beltramello M, Espinosa DA, et al. Specificity, cross-reactivity, and function of antibodies elicited by Zika virus infection. Science. 2016;353:823–826. doi: 10.1126/science.aaf8505 27417494

[24] Wang Q, Yang H, Liu X, et al. Molecular determinants of human neutralizing antibodies isolated from a patient infected with Zika virus. Sci Transl Med. 2016;8:369ra179–369ra179. doi: 10.1126/scitranslmed.aai8336 27974667

[25] Yu L, Wang R, Gao F, et al. Delineating antibody recognition against Zika virus during natural infection. JCI Insight. 2017;2:e93042. doi: 10.1172/jci.insight.93042 PMC5470883 28614803

[26] Zhang F-C, Li X-F, Deng Y-Q, et al. Excretion of infectious Zika virus in urine. Lancet Infect Dis. 2016;16:641–642. doi: 10.1016/S1473-3099(16)30070-6 27184420

[27] Wu X, Zhang Z, Schramm CA, et al. Maturation and diversity of the VRC01-antibody lineage over 15 years of chronic HIV-1 infection. Cell. 2015;161:470–485. doi: 10.1016/j.cell.2015.03.004 25865483 PMC4706178

[28] Liao H-X, Levesque MC, Nagel A, et al. High-throughput isolation of immunoglobulin genes from single human B cells and expression as monoclonal antibodies. J Virol Methods. 2009;158:171–179. doi: 10.1016/j.jviromet.2009.02.014 19428587 PMC2805188

[29] Brochet X, Lefranc M-PP, Giudicelli V. IMGT/V-QUEST: The highly customized and integrated system for IG and TR standardized V-J and V-D-J sequence analysis. Nucleic Acids Res. 2008;36:W503–W508. doi: 10.1093/nar/gkn316 18503082 PMC2447746

[30] Meng W, Pan W, Zhang AJ, et al*.* Rapid generation of human-like neutralizing monoclonal antibodies in urgent preparedness for influenza pandemics and virulent infectious diseases. PLoS ONE. 2013;8:e66276. doi: 10.1371/journal.pone.0066276 23824680 PMC3688872

[31] Kraus AA, Messer W, Haymore LB, et al. Comparison of plaque- and flow cytometry-based methods for measuring dengue virus neutralization. J Clin Microbiol. 2007;45:3777–3780. doi: 10.1128/JCM.00827-07 17804661 PMC2168473

[32] Davidson E, Doranz BJ. A high-throughput shotgun mutagenesis approach to mapping B-cell antibody epitopes. Immunology. 2014;143:13–20. doi: 10.1111/imm.12323 24854488 PMC4137951

[33] Dejnirattisai W, Wongwiwat W, Supasa S, et al. A new class of highly potent, broadly neutralizing antibodies isolated from viremic patients infected with dengue virus. Nature Immunol. 2015;16:170–177. doi: 10.1038/ni.3058 25501631 PMC4445969

[34] Wrammert J, Smith K, Miller J, et al. Rapid cloning of high-affinity human monoclonal antibodies against influenza virus. Nature. 2008;453:667–671. doi: 10.1038/nature06890 18449194 PMC2515609

[35] Zhou T, Lynch RM, Chen L, et al. Structural repertoire of HIV-1-neutralizing antibodies targeting the CD4 supersite in 14 donors. Cell. 2015;161:1280–1292. doi: 10.1016/j.cell.2015.05.007 26004070 PMC4683157

[36] Corti D, Zhao J, Pedotti M, et al. Prophylactic and postexposure efficacy of a potent human monoclonal antibody against MERS coronavirus. Proc Natl Acad Sci USA. 2015;112:10473–10478. doi: 10.1073/pnas.1510199112 26216974 PMC4547275

[37] Ying T, Du L, Ju T, et al. Exceptionally potent neutralization of Middle East respiratory syndrome coronavirus by human monoclonal antibodies. J Virol. 2014;88:7796–7805. doi: 10.1128/JVI.00912-14 24789777 PMC4097770

[38] Zhang Q, Gui M, Niu X, et al. Potent neutralizing monoclonal antibodies against Ebola virus infection. Sci Rep. 2016;6:25856. doi: 10.1038/srep25856 27181584 PMC4867612

[39] Wang J, Bardelli M, Espinosa DA, et al. A human bi-specific antibody against Zika virus with high therapeutic potential. Cell. 2017;171:229–241.e15. doi: 10.1016/j.cell.2017.09.002 28938115 PMC5673489

[40] Magnani DM, Rogers TF, Beutler N, et al*.* Neutralizing human monoclonal antibodies prevent Zika virus infection in macaques. Sci Transl Med. 2017;9:eaan8184. doi: 10.1126/scitranslmed.aan8184 28978754 PMC6155977

[41] Wang Q, Yan J, Gao GF. Monoclonal antibodies against Zika virus: therapeutics and their implications for vaccine design. J Virol. 2017;91:e01049–17. 28768876 10.1128/JVI.01049-17 PMC5625485

[42] Zhao H, Fernandez E, Dowd KA, et al. Structural basis of Zika virus-specific antibody protection. Cell. 2016;166:1016–1027. doi: 10.1016/j.cell.2016.07.020 27475895 PMC4983199

[43] Deng Y-QQ, Dai J-XX, Ji G-HH, et al*.* A broadly flavivirus cross-neutralizing monoclonal antibody that recognizes a novel epitope within the fusion loop of E protein. PloS ONE. 2011;6:e16059. doi: 10.1371/journal.pone.0016059 21264311 PMC3019176

[44] Dejnirattisai W, Supasa P, Wongwiwat W, et al. Dengue virus sero-cross-reactivity drives antibody-dependent enhancement of infection with zika virus. Nature Immunol. 2016;17:1102–1108. doi: 10.1038/ni.3515 27339099 PMC4994874

[45] Abbink P, Larocca RA, Dejnirattisai W, et al. Therapeutic and protective efficacy of a dengue antibody against Zika infection in rhesus monkeys. Nature Med. 2018;24:721–723. doi: 10.1038/s41591-018-0056-0 29867228 PMC5992501

[46] Fernandez E, Dejnirattisai W, Cao B, et al. Human antibodies to the dengue virus E-dimer epitope have therapeutic activity against Zika virus infection. Nature Immunol. 2017;18:1261–1269. doi: 10.1038/ni.3849 28945244 PMC5679314

[47] Hasan S, Miller A, Sapparapu G, et al*.* A human antibody against Zika virus crosslinks the E protein to prevent infection. Nature Comm. 2017;8:14722. doi: 10.1038/ncomms14722 PMC5356071 28300075

[48] Long F, Doyle M, Fernandez E, et al. Structural basis of a potent human monoclonal antibody against Zika virus targeting a quaternary epitope. Proc Natl Acad Sci USA. 2019;116:1591–1596. doi: 10.1073/pnas.1815432116 30642974 PMC6358714

[49] Lazear HM, Govero J, Smith A, et al. A mouse model of Zika virus pathogenesis. Cell Host Microbe. 2016;19:720–730. doi: 10.1016/j.chom.2016.03.010 27066744 PMC4866885

[50] Manangeeswaran M, Ireland DD, Verthelyi D. Zika (PRVABC59) infection is associated with T cell infiltration and neurodegeneration in CNS of immunocompetent neonatal C57Bl/6 mice. PLoS Pathog. 2016;12:e1006004. doi: 10.1371/journal.ppat.1006004 27855206 PMC5113993

[51] Aliota MT, Caine EA, Walker EC, et al. Characterization of lethal Zika virus infection in AG129 mice. PLOS Negl Trop Dis. 2016;10:e0004682. doi: 10.1371/journal.pntd.0004682 27093158 PMC4836712

